# Supplementary material for: Impact of digital health interventions on pain and symptom management in home hospice patients: A systematic review and meta-analysis protocol
Source: PLoS One. 2025 Oct 9;20(10):e0333513. doi: 10.1371/journal.pone.0333513 (PMC12510486; doi:10.1371/journal.pone.0333513)
Supplement: S1 File — (DOCX) [file pone.0333513.s001.docx]

**Peer Review of Electronic Search Strategies**

***PRESS Guideline* — Search Submission & Peer Review Assessment SEARCH SUBMISSION:**

| Searcher: Thiago Oliveira dos Santos | Email: enf.thiagoliveira@gmail.com |
| --- | --- |
| Date submitted: | Date requested by: |

**Systematic Review Title:**

| **“MPACT OF DIGITAL HEALTH USED FOR PAIN MANAGEMENT AND OTHERS SYMPTOMS OF PATIENTS WHO ARE RECEIVING HOME HOSPICE CARE: a systematic review and Analysis protocol”** |
| --- |

**This search strategy is…**

| X | My PRIMARY (core) database strategy — First time submitting a strategy for search question and database |
| --- | --- |
|  | My PRIMARY (core) strategy — Follow-up review NOT the first time submitting a strategy for search question and database. If this is a response to peer review, itemize the changes made to the review suggestions |
|  | SECONDARY search strategy— First time submitting a strategy for search question and database |
|  | SECONDARY search strategy — NOT the first time submitting a strategy for search question and database. If this is a response to peer review, itemize the changes made to the review suggestions |

**Database**

(i.e., MEDLINE, CINAHL…): *[mandatory]*

| *CINAHL (EBSCO), Cochrane Library, Embase (Elsevier), LILACS, PubMed/MEDLINE, Scopus (Elsevier), Web of Science (Clarivate Analytics), Scielo* |
| --- |

**Interface**

(i.e., Ovid, EBSCO…): *[mandatory]*

| PubMed |
| --- |

**Research Question**

(Describe the purpose of the search) *[mandatory]*

| What is the effectiveness of telemedicine in pain management for cancer patients in palliative care compared to traditional in-person care? |
| --- |

**PICO Format**

(Outline the PICOs for your question — i.e., Patient, Intervention, Comparison, Outcome, and Study Design — as applicable)

| **P** | Palliative Care Patients |
| --- | --- |
| **I** | Telemedicine / Digital Health |
| **C** | In-Person Outpatient Care |
| **O** | Management of Signs and Symptoms (Pain) Quasi-Experimental Studies |

**Inclusion Criteria**

(List criteria such as age groups, study designs, etc., to be included) *[optional]*

| This systematic review will include observational studies, such as case-control and cohort studies, involving adult oncology patients treated through digital health tools. Studies published in any language and year will be considered, provided they have undergone peer review, thereby excluding publications that have not undergone this process. |
| --- |

**Exclusion Criteria**

(List criteria such as study designs, date limits, etc., to be excluded) *[optional]*

| Case reports, observational, retrospective studies, cohort studies, case reports, observational, retrospective studies, cohort studies |
| --- |

**Was a search filter applied?**

Yes **X** No □

**If YES, which one(s) (e.g., Cochrane RCT filter, PubMed Clinical Queries filter)? Provide the source if this is a published filter.** *[mandatory if YES to previous question* — *textbox]*

| *In the PUBMED database, brackets and parentheses " " were used in compound words. EX: (Pain) AND (Palliative Care)]*  *In the SCOPUS database, Pain Surgery was used as a descriptor* |
| --- |

Other notes or comments you feel would be useful for the peer reviewer? ***[optional]***

|  |
| --- |

Please copy and paste your search strategy here, exactly as run, including the number of hits per line. ***[mandatory]***

**(Add more space, as necessary.)**

|  | **DECS or MESH and synonyms** | **STRATEGY LINES** | **NUMBER OF STUDIES LOCATED** |
| --- | --- | --- | --- |
| **P** | Palliative Care  Palliative | ("Palliative Care"[Mesh] OR "Palliative Care" OR "Palliative") | 138.668 |
| **AND** | | | |
| **I** | Cyber Health Cyber-Health Cyberhealth e-Health eHealth Medicine 2.0 Virtual Medicine mHealth Health 2.0 Connected Health Electronic Health Mobile Health Telehealth Tele-Services in Health Telecare Telehealth Services Telehealth Services in Health Telehealth Services in Healthcare | ("Telemedicine"[Mesh] OR "Telemedicine" OR "Connected Health" OR "eHealth" OR "Health 2.0" OR "Health Tele-Services" OR "Health Teleservices" OR "Medicine 2.0" OR "mHealth" OR "Mobile Health" OR "Tele Care" OR "Tele-Care" OR "Telecare" OR "Telecure" OR "Telehealth" OR "Teleservices in the Health Sector" OR "Virtual Medicine") | 97.817 |
| **AND** | | | |
| **C** |  |  |  |
| **AND** | | | |
| **O** |  |  |  |
| **STRATEGY** | | | |
| ("Pain"[Mesh] OR "Pain" OR "Pains" OR "Physical Suffering" OR "Physical Sufferings" OR "Ache" OR "Aches") AND ("Palliative Care"[Mesh] OR "Palliative Care" OR "Palliative") AND ("Telemedicine"[Mesh] OR "Telemedicine" OR "Connected Health" OR "eHealth" OR "Health 2.0" OR "Health Tele-Services" OR "Health Teleservices" OR "Medicine 2.0" OR "mHealth" OR "Mobile Health" OR "Tele Care" OR "Tele-Care" OR "Telecare" OR "Telecure" OR "Telehealth" OR "Teleservices in the Health Sector" OR "Virtual Medicine") | | | |

* Use adaptations of the acronym as needed.

| **DATABASE** | **STRATEGY 1** | **NUMBER OF STUDIES LOCATED** |
| --- | --- | --- |
| PubMed/Medline | ("Pain"[Mesh] OR "Pain" OR "Pains" OR "Physical Suffering" OR "Physical Sufferings" OR "Ache" OR "Aches") AND ("Palliative Care"[Mesh] OR "Palliative Care" OR "Palliative") AND ("Telemedicine"[Mesh] OR "Telemedicine" OR "Connected Health" OR "eHealth" OR "Health 2.0" OR "Health Tele-Services" OR "Health Teleservices" OR "Medicine 2.0" OR "mHealth" OR "Mobile Health" OR "Tele Care" OR "Tele-Care" OR "Telecare" OR "Telecure" OR "Telehealth" OR "Teleservices in the Health Sector" OR "Virtual Medicine") | 244 |
| Embase | ("Pain" OR "Pains" OR "Physical Suffering" OR "Physical Sufferings" OR "Ache" OR "Aches") AND ("Palliative Care" OR "Palliative") AND ("Telemedicine" OR "Connected Health" OR "eHealth" OR "Health 2.0" OR "Health Tele-Services" OR "Health Teleservices" OR "Medicine 2.0" OR "mHealth" OR "Mobile Health" OR "Tele Care" OR "Tele-Care" OR "Telecare" OR "Telecure" OR "Telehealth" OR "Teleservices in the Health Sector" OR "Virtual Medicine") | 348 |
| CINAHL (EBSCO | ("Pain" OR "Pains" OR "Physical Suffering" OR "Physical Sufferings" OR "Ache" OR "Aches") AND ("Palliative Care" OR "Palliative") AND ("Telemedicine" OR "Connected Health" OR "eHealth" OR "Health 2.0" OR "Health Tele-Services" OR "Health Teleservices" OR "Medicine 2.0" OR "mHealth" OR "Mobile Health" OR "Tele Care" OR "Tele-Care" OR "Telecare" OR "Telecure" OR "Telehealth" OR "Teleservices in the Health Sector" OR "Virtual Medicine") | 63 |
| Cochrane | ("Pain" OR "Pains" OR "Physical Suffering" OR "Physical Sufferings" OR "Ache" OR "Aches") AND ("Palliative Care" OR "Palliative") AND ("Telemedicine" OR "Connected Health" OR "eHealth" OR "Health 2.0" OR "Health Tele-Services" OR "Health Teleservices" OR "Medicine 2.0" OR "mHealth" OR "Mobile Health" OR "Tele Care" OR "Tele-Care" OR "Telecare" OR "Telecure" OR "Telehealth" OR "Teleservices in the Health Sector" OR "Virtual Medicine") | 42 |
| Scopus (Elsevier) | ("Pain" OR "Pains" OR "Physical Suffering" OR "Physical Sufferings" OR "Ache" OR "Aches") AND ("Palliative Care" OR "Palliative") AND ("Telemedicine" OR "Connected Health" OR "eHealth" OR "Health 2.0" OR "Health Tele-Services" OR "Health Teleservices" OR "Medicine 2.0" OR "mHealth" OR "Mobile Health" OR "Tele Care" OR "Tele-Care" OR "Telecare" OR "Telecure" OR "Telehealth" OR "Teleservices in the Health Sector" OR "Virtual Medicine") | 188 |
| Web of Science | ("Pain" OR "Pains" OR "Physical Suffering" OR "Physical Sufferings" OR "Ache" OR "Aches") AND ("Palliative Care" OR "Palliative") AND ("Telemedicine" OR "Connected Health" OR "eHealth" OR "Health 2.0" OR "Health Tele-Services" OR "Health Teleservices" OR "Medicine 2.0" OR "mHealth" OR "Mobile Health" OR "Tele Care" OR "Tele-Care" OR "Telecare" OR "Telecure" OR "Telehealth" OR "Teleservices in the Health Sector" OR "Virtual Medicine") | 242 |
| LILACS | ("Pain" OR "Pains" OR "Physical Suffering" OR "Physical Sufferings" OR "Ache" OR "Aches" OR "Dor" OR "Algia" OR "Sensação de Ardência" OR "Sofrimento Físico" OR "Dolor" OR "sufrimiento físico") AND ("Palliative Care" OR "Palliative" OR "Cuidados Paliativos" OR Paliativ*) AND ("Telemedicine" OR "Connected Health" OR "eHealth" OR "Health 2.0" OR "Health Tele-Services" OR "Health Teleservices" OR "Medicine 2.0" OR "mHealth" OR "Mobile Health" OR "Tele Care" OR "Tele-Care" OR "Telecare" OR "Telecure" OR "Telehealth" OR "Teleservices in the Health Sector" OR "Virtual Medicine" OR "Telemedicina" OR "Ciber Saúde" OR "Ciber-Saúde" OR "Cibersaúde" OR "e-Saúde" OR "eSaúde" OR "Medicina 2.0" OR "Medicina Virtual" OR "mSaúde" OR "Saúde 2.0" OR "Saúde Conectada" OR "Saúde Eletrônica" OR "Saúde Móvel" OR "Telessaúde" OR "Tele-Serviços em Saúde" OR "Teleassistência" OR "Telesserviços de Saúde" OR "Telesserviços em Saúde" OR "Telesserviços na Saúde" OR "eSalud" OR "Ciber Salud" OR "Ciber-Salud" OR "Cibersalud" OR "mSalud" OR "Salud 2.0" OR "Salud Conectada" OR "Salud Electrónica" OR "Salud Móvil" OR "Salud Mueble" OR "Telesalud" OR "Teleasistencia" OR "Telesalud" OR "Teleservicios de Salud") | 28 |
| Scielo | ("Pain" OR "Pains" OR "Physical Suffering" OR "Physical Sufferings" OR "Ache" OR "Aches" OR "Dor" OR "Algia" OR "Sensação de Ardência" OR "Sofrimento Físico" OR "Dolor" OR "sufrimiento físico") AND ("Palliative Care" OR "Palliative" OR "Cuidados Paliativos" OR Paliativ*) AND ("Telemedicine" OR "Connected Health" OR "eHealth" OR "Health 2.0" OR "Health Tele-Services" OR "Health Teleservices" OR "Medicine 2.0" OR "mHealth" OR "Mobile Health" OR "Tele Care" OR "Tele-Care" OR "Telecare" OR "Telecure" OR "Telehealth" OR "Teleservices in the Health Sector" OR "Virtual Medicine" OR "Telemedicina" OR "Ciber Saúde" OR "Ciber-Saúde" OR "Cibersaúde" OR "e-Saúde" OR "eSaúde" OR "Medicina 2.0" OR "Medicina Virtual" OR "mSaúde" OR "Saúde 2.0" OR "Saúde Conectada" OR "Saúde Eletrônica" OR "Saúde Móvel" OR "Telessaúde" OR "Tele-Serviços em Saúde" OR "Teleassistência" OR "Telesserviços de Saúde" OR "Telesserviços em Saúde" OR "Telesserviços na Saúde" OR "eSalud" OR "Ciber Salud" OR "Ciber-Salud" OR "Cibersalud" OR "mSalud" OR "Salud 2.0" OR "Salud Conectada" OR "Salud Electrónica" OR "Salud Móvil" OR "Salud Mueble" OR "Telesalud" OR "Teleasistencia" OR "Telesalud" OR "Teleservicios de Salud") | 05 |

**PEER REVIEW ASSESSMENT: THIS SECTION TO BE FILLED IN BY THE REVIEWER**

| **Reviewer:** | **Email:** | | **Date completed:** |
| --- | --- | --- | --- |
| **1. TRANSLATION** | | | |
|  | A. No revisions | **□** |  |
|  | B. Revision(s) suggested | **□** |  |
|  | C. Revision(s) required | **□** |  |

If “B” or “C,” please provide an explanation or example:

|  |
| --- |

| **2. BOOLEAN AND PROXIMITY OPERATORS** | | | |
| --- | --- | --- | --- |
|  | A. No revisions | **□** |  |
|  | B. Revision(s) suggested | **□** |  |
|  | C. Revision(s) required | **□** |  |

If “B” or “C,” please provide an explanation or example:

|  |
| --- |

| **3. SUBJECT HEADINGS** | | | |
| --- | --- | --- | --- |
|  | A. No revisions | **□** |  |
|  | B. Revision(s) suggested | **□** |  |
|  | C. Revision(s) required | **□** |  |

If “B” or “C,” please provide an explanation or example:

|  |
| --- |

| **4. TEXT WORD SEARCHING** | | | |
| --- | --- | --- | --- |
|  | A. No revisions | **□** |  |
|  | B. Revision(s) suggested | **□** |  |
|  | C. Revision(s) required | **□** |  |

If “B” or “C,” please provide an explanation or example:

|  |
| --- |

| **5. SPELLING, SYNTAX, AND LINE NUMBERS** | | | |
| --- | --- | --- | --- |
|  | A. No revisions | **□** |  |
|  | B. Revision(s) suggested | **□** |  |
|  | C. Revision(s) required | **□** |  |

If “B” or “C,” please provide an explanation or example:

|  |
| --- |

| **6. LIMITS AND FILTERS** | | | |
| --- | --- | --- | --- |
|  | A. No revisions | **□** |  |
|  | B. Revision(s) suggested | **□** |  |
|  | C. Revision(s) required | **□** |  |

If “B” or “C,” please provide an explanation or example:

|  |
| --- |

| **7. OVERALL EVALUATION (Note: If one or more “revision required” is noted above, the**  **response below must be “revisions required”.)** | | | |
| --- | --- | --- | --- |
|  | A. No revisions | **□** |  |
|  | B. Revision(s) suggested | **□** |  |
|  | C. Revision(s) required | **□** |  |

Additional comments:

|  |
| --- |

## History and Search Details

窗体顶端

Download

窗体底端

Delete

| **Search** | **Actions** | **Details** | **Query** | **Results** | **Time** |
| --- | --- | --- | --- | --- | --- |
| #23 |  |  | Search: **((((Patient) OR (Patiens)) AND ((Adult) OR (Adults))) AND (((((((((((Anemia) OR (Anemias)) OR (Anemia, Iron-Deficiency)) OR (Anemias, Iron-Deficiency)) ) OR (Anemia, Iron Deficiency)) OR (Anemias, Iron Deficiency )OR (Iron Deficiencies)) OR (Iron Deficiency Anemia)) OR (Iron Deficiency Anemias)) OR (Iron-Deficiency Anemia)) OR (Iron-Deficiency Anemias))) AND (((((((((((((Ferric Oxide, Saccharated) OR (Ferrous sulfate)) OR (Intravenous iron)) OR (IV iron)) OR (Ferric carboxymaltose)) OR (Iron sucrose)) OR (Ferric gluconate)) OR (Iron isomaltoside)) OR (Ferric derisomaltose)) OR (Iron hydroxide)) OR (Iron supplementation)) AND (((((((Preoperative Period) OR (Period, Preoperative)) OR (Preoperative Care)) OR (Perioperative Period)) OR (Period, Perioperative)) OR (Periods, Perioperative)) OR (Perioperative Periods))) AND ((Cardiac Surgery) OR (Heart Surgery) OR (Surgery Cardiac) OR (Surgery, Heart) OR (Coronary Artery Bypass Graft) OR (CABG) OR (Valve surgery) OR (Extracorporeal circulation)))** | [38](https://pubmed.ncbi.nlm.nih.gov/?term=((((Patient)+OR+(Patiens))+AND+((Adult)+OR+(Adults)))+AND+(((((((((((Anemia)+OR+(Anemias))+OR+(Anemia,+Iron-Deficiency))+OR+(Anemias,+Iron-Deficiency))+)+OR+(Anemia,+Iron+Deficiency))+OR+(Anemias,+Iron+Deficiency+)OR+(Iron+Deficiencies))+OR+(Iron+Deficiency+Anemia))+OR+(Iron+Deficiency+Anemias))+OR+(Iron-Deficiency+Anemia))+OR+(Iron-Deficiency+Anemias)))+AND+(((((((((((((Ferric+Oxide,+Saccharated)+OR+(Ferrous+sulfate))+OR+(Intravenous+iron))+OR+(IV+iron))+OR+(Ferric+carboxymaltose))+OR+(Iron+sucrose))+OR+(Ferric+gluconate))+OR+(Iron+isomaltoside))+OR+(Ferric+derisomaltose))+OR+(Iron+hydroxide))+OR+(Iron+supplementation))+AND+(((((((Preoperative+Period)+OR+(Period,+Preoperative))+OR+(Preoperative+Care))+OR+(Perioperative+Period))+OR+(Period,+Perioperative))+OR+(Periods,+Perioperative))+OR+(Perioperative+Periods)))+AND+((Cardiac+Surgery)+OR+(Heart+Surgery)+OR+(Surgery+Cardiac)+OR+(Surgery,+Heart)+OR+(Coronary+Artery+Bypass+Graft)+OR+(CABG)+OR+(Valve+surgery)+OR+(Extracorporeal+circulation)))&sort=) | 12:00:07 |
| #28 |  |  | Search: **((((Patient) OR (Patiens)) AND ((Adult) OR (Adults))) AND (((((((((((Anemia) OR (Anemias)) OR ("Anemia, Iron-Deficiency")) OR ("Anemias, Iron-Deficiency")) ) OR ("Anemia, Iron Deficiency")) OR ("Anemias, Iron Deficiency") OR ("Iron Deficiencies")) OR ("Iron Deficiency Anemia")) OR ("Iron Deficiency Anemias")) OR ("Iron-Deficiency Anemia")) OR ("Iron-Deficiency Anemias"))) AND ((((((((((((("Ferric Oxide, Saccharated") OR ("Ferrous sulfate")) OR ("Intravenous iron")) OR ("IV iron")) OR ("Ferric carboxymaltose")) OR ("Iron sucrose")) OR ("Ferric gluconate")) OR ("Iron isomaltoside")) OR ("Ferric derisomaltose")) OR ("Iron hydroxide")) OR ("Iron supplementation")) AND ((((((("Preoperative Period") OR ("Period, Preoperative")) OR ("Preoperative Care")) OR ("Perioperative Period")) OR ("Period, Perioperative")) OR ("Periods, Perioperative")) OR ("Perioperative Periods"))) AND (("Cardiac Surgery") OR ("Heart Surgery") OR ("Surgery Cardiac") OR ("Surgery, Heart") OR ("Coronary Artery Bypass Graft") OR (CABG) OR ("Valve surgery") OR ("Extracorporeal circulation")))** | [12](https://pubmed.ncbi.nlm.nih.gov/?term=%C2%A0((((Patient)+OR+(Patiens))+AND+((Adult)+OR+(Adults)))+AND+(((((((((((Anemia)+OR+(Anemias))+OR+(%E2%80%9CAnemia,+Iron-Deficiency%E2%80%9C))+OR+(%E2%80%9CAnemias,+Iron-Deficiency%E2%80%9C))+)+OR+(%E2%80%9CAnemia,+Iron+Deficiency%E2%80%9C))+OR+(%E2%80%9CAnemias,+Iron+Deficiency%E2%80%9C)+OR+(%E2%80%9CIron+Deficiencies%E2%80%9C))+OR+(%E2%80%9CIron+Deficiency+Anemia%E2%80%9C))+OR+(%E2%80%9CIron+Deficiency+Anemias%E2%80%9C))+OR+(%E2%80%9CIron-Deficiency+Anemia%E2%80%9C))+OR+(%E2%80%9CIron-Deficiency+Anemias%E2%80%9C)))+AND+(((((((((((((%E2%80%9CFerric+Oxide,+Saccharated%E2%80%9C)+OR+(%E2%80%9CFerrous+sulfate%E2%80%9C))+OR+(%E2%80%9CIntravenous+iron%E2%80%9C))+OR+(%E2%80%9CIV+iron%E2%80%9C))+OR+(%E2%80%9CFerric+carboxymaltose%E2%80%9C))+OR+(%E2%80%9CIron+sucrose%E2%80%9C))+OR+(%E2%80%9CFerric+gluconate%E2%80%9C))+OR+(%E2%80%9CIron+isomaltoside%E2%80%9C))+OR+(%E2%80%9CFerric+derisomaltose%E2%80%9C))+OR+(%E2%80%9CIron+hydroxide%E2%80%9C))+OR+(%E2%80%9CIron+supplementation%E2%80%9C))+AND+(((((((%E2%80%9CPreoperative+Period%E2%80%9C)+OR+(%E2%80%9CPeriod,+Preoperative%E2%80%9C))+OR+(%E2%80%9CPreoperative+Care%E2%80%9C))+OR+(%E2%80%9CPerioperative+Period%E2%80%9C))+OR+(%E2%80%9CPeriod,+Perioperative%E2%80%9C))+OR+(%E2%80%9CPeriods,+Perioperative%E2%80%9C))+OR+(%E2%80%9CPerioperative+Periods%E2%80%9C)))+AND+((%E2%80%9CCardiac+Surgery%E2%80%9C)+OR+(%E2%80%9CHeart+Surgery%E2%80%9C)+OR+(%E2%80%9CSurgery+Cardiac%E2%80%9C)+OR+(%E2%80%9CSurgery,+Heart%E2%80%9C)+OR+(%E2%80%9CCoronary+Artery+Bypass+Graft%E2%80%9C)+OR+(CABG)+OR+(%E2%80%9CValve+surgery%E2%80%9C)+OR+(%E2%80%9CExtracorporeal+circulation%E2%80%9C)))&sort=) | 11:58:53 |
| #20 |  |  | Search: **((((((((((((Ferric Oxide, Saccharated) OR (Ferrous sulfate)) OR (Intravenous iron)) OR (IV iron)) OR (Ferric carboxymaltose)) OR (Iron sucrose)) OR (Ferric gluconate)) OR (Iron isomaltoside)) OR (Ferric derisomaltose)) OR (Iron hydroxide)) OR (Iron supplementation)) AND (((((((Preoperative Period) OR (Period, Preoperative)) OR (Preoperative Care)) OR (Perioperative Period)) OR (Period, Perioperative)) OR (Periods, Perioperative)) OR (Perioperative Periods))) AND ((Cardiac Surgery) OR (Heart Surgery) OR (Surgery Cardiac) OR (Surgery, Heart) OR (Coronary Artery Bypass Graft) OR (CABG) OR (Valve surgery) OR (Extracorporeal circulation))** | [90](https://pubmed.ncbi.nlm.nih.gov/?term=((((((((((((Ferric+Oxide,+Saccharated)+OR+(Ferrous+sulfate))+OR+(Intravenous+iron))+OR+(IV+iron))+OR+(Ferric+carboxymaltose))+OR+(Iron+sucrose))+OR+(Ferric+gluconate))+OR+(Iron+isomaltoside))+OR+(Ferric+derisomaltose))+OR+(Iron+hydroxide))+OR+(Iron+supplementation))+AND+(((((((Preoperative+Period)+OR+(Period,+Preoperative))+OR+(Preoperative+Care))+OR+(Perioperative+Period))+OR+(Period,+Perioperative))+OR+(Periods,+Perioperative))+OR+(Perioperative+Periods)))+AND+((Cardiac+Surgery)+OR+(Heart+Surgery)+OR+(Surgery+Cardiac)+OR+(Surgery,+Heart)+OR+(Coronary+Artery+Bypass+Graft)+OR+(CABG)+OR+(Valve+surgery)+OR+(Extracorporeal+circulation))&sort=) | 10:16:55 |
| #5 |  |  | Search: **(((Patient) OR (Patiens)) AND ((Adult) OR (Adults))) AND (((((((((((Anemia) OR (Anemias)) OR (Anemia, Iron-Deficiency)) OR (Anemias, Iron-Deficiency)) ) OR (Anemia, Iron Deficiency)) OR (Anemias, Iron Deficiency )OR (Iron Deficiencies)) OR (Iron Deficiency Anemia)) OR (Iron Deficiency Anemias)) OR (Iron-Deficiency Anemia)) OR (Iron-Deficiency Anemias))** | [68,143](https://pubmed.ncbi.nlm.nih.gov/?term=(((Patient)+OR+(Patiens))+AND+((Adult)+OR+(Adults)))+AND+(((((((((((Anemia)+OR+(Anemias))+OR+(Anemia,+Iron-Deficiency))+OR+(Anemias,+Iron-Deficiency))+)+OR+(Anemia,+Iron+Deficiency))+OR+(Anemias,+Iron+Deficiency+)OR+(Iron+Deficiencies))+OR+(Iron+Deficiency+Anemia))+OR+(Iron+Deficiency+Anemias))+OR+(Iron-Deficiency+Anemia))+OR+(Iron-Deficiency+Anemias))&sort=) | 09:35:07 |

窗体底端
